# Atomic Force Microscope Study of Ag-Conduct Polymer Hybrid Films: Evidence for Light-Induced Charge Separation

**DOI:** 10.3390/nano10091819

**Published:** 2020-09-12

**Authors:** Yinghui Wu, Dong Wang, Jinyuan Liu, Houzhi Cai, Yueqiang Zhang

**Affiliations:** Key Laboratory of Optoelectronic Devices and Systems of Ministry of Education and Guangdong Province, College of Physics and Optoelectronic Engineering, Shenzhen University, Shenzhen 518060, China; yinghui@szu.edu.cn (Y.W.); wangdong20@szu.edu.cn (D.W.); ljy@szu.edu.cn (J.L.); hzcai@szu.edu.cn (H.C.)

**Keywords:** SKPM, EFM, conductive polymers, interface

## Abstract

Scanning Kelvin probe microscopy (SKPM), electrostatic force microscopy (EFM) are used to study the microscopic processes of the photo-induced charge separation at the interface of Ag and conductive polymers, i.e., poly[2,6-(4,4-bis-(2-ethylhexyl)-4*H*-cyclopenta[2,1-b;3,4-bʹ]dithiophene)-alt-4,7-(2,1,3-benzothiadiazole)] (PCPDTBT) and poly(3-hexylthiophene-2,5-diyl) (P3HT). They are also widely used in order to directly observe the charge distribution and dynamic changes at the interfaces in nanostructures, owing to their high sensitivity. Using SKPM, it is proved that the charge of the photo-induced polymer PCPDTBT is transferred to Ag nanoparticles (NPs). The surface charge of the Ag-induced NPs is quantified while using EFM, and it is determined that the charge is injected into the polymer P3HT from the Ag NPs. We expect that this technology will provide guidance to facilitate the separation and transfer of the interfacial charges in the composite material systems and it will be applicable to various photovoltaic material systems.

## 1. Introduction

Noble metal nanoparticles (NPs) not only increase light absorption owing to their local surface plasmon effect, strong scattering effect, and diversity in size and morphology, but also exhibit enhanced electrical, optical, and magnetic properties [1,2,3,4]. Therefore, NPs have a wide range of applications in Light Emitting Diode (LED) technology, microscopy technology, data storage, and biomedical fields [5,6,7,8,9,10,11]. Conductive polymers have always been of interest to researchers, owing to their relatively unique properties, such as light weight, good toughness, easy processing, easy conductivity adjustment, low cost, easy large-area coating, and convenient fabrication. They have both electrical and optical properties of metals and inorganic semiconductors, the flexible mechanical properties and processability of organic polymers, and electrochemical redox activity [12,13,14]. It is possible to combine the abovementioned advantages by integrating precious metals with conductive polymers and designing composite nanostructures; their application in solar photovoltaic materials has attracted widespread attention, owing to their unique interface charge separation performance [15,16,17]. To date, noble metal NPs mixed with conductive polymers, e.g., poly(3-hexylthiophene-2,5-diyl) (P3HT), (6,6)-phenyl-C61-butyric acid methyl ester, poly(2-methoxy-5-(2-ethylhexyloxy)-1,4-phenylenevinylene) (MEH-PPV), and poly(3,4-ethylenedioxythiophene)-poly(styrene sulfonate) (PEDOT:PSS), have been applied to fabrication of photovoltaic solar cells [18,19,20]. The interface charge separation process largely affects the conversion efficiency of these noble metal-polymer composite-nanostructure photovoltaic solar cells.

It is particularly important to track every step in the photoelectric conversion process from a microscopic perspective. However, it is difficult to achieve this requirement through conventional technical means. The charge distribution of nanostructures and the local electrostatic potential of the sample surface can be effectively measured by electrostatic force microscopy (EFM) and scanning Kelvin probe microscopy (scale) [21,22,23] (Figure S1). For example, it is found via SKPM that charge separation at the interface between the Au and TiO_2_ NPs varied under UV and visible-near infrared light [24]. The charge injection and transport in the highly oriented P3HT film induced by nano-CdS/CdSe have been reported [22]. The charge separation at the interface of MEH-PPV and TiO_2_ nanocomposite film have been also directly observed [23]. EFM has been used to investigate the charge state of single NPs [21,25,26]. However, there are few reports on the study of charge transfer at the interface between noble metal and polymers through SKPM and EFM.

In this work, the changes in surface charge of Ag NPs on different substrates before and after illumination were investigated through SKPM and EFM. First, the size of Ag NPs was characterized via transmission electron microscopy (TEM) and atomic force microscopy (AFM). Subsequently, the plasmon resonance frequency of Ag NPs was measured using UV-visible spectra, and the surface plasmon of Ag NPs in conductive polymers was confirmed via surface-enhanced Raman scattering of the vibrational spectroscopy. The SKPM and EFM modes were used to quantitatively study the light-modulated surface potentials of Ag NPs and the photo-induced charge separation/electron transfer process between Ag NPs and different substrates. Specific processes utilize the EFM mode for measuring the electron number of Ag NPs, elucidating the influence of Ag NP surface plasmon polaritons on different substrates, and demonstrating the process of light-induced charge separation. 

## 2. Materials and Methods

Poly(3-hexylthiophene-2,5-diyl) (P3HT), poly[2,6-(4,4-bis-(2-ethylhexyl)-4*H*-cyclopenta[2,1-b;3,4-b′]dithiophene)-alt-4,7-(2,1,3-benzothiadiazole)] (PCPDTBT), and all solvents (chloroform, chlorobenzene) were purchased from Sigma Aldrich (Beijing, China). The ITO (indium tin oxide) glass substrates (100 mm × 100 mm × 1.1 mm, 8 Ω/square) and FTO (fluorine-doped tin oxide) glass substrates (100 mm × 100 mm × 2.2 mm, 14 Ω/square) were purchased from Sigma Aldrich. All of the solvents were purchased from Acros Organic (Shanghai, China) and Sigma-Aldrich and used as obtained.

### 2.1. Synthesis of Ag Nano Particles (NPs)

To synthesize Ag NPs, PPV and AgNO_3_ were dissolved into 5 mL of ethylene glycol solution with continuous stirring in an oil bath (Partulab, Wuhan, China), at 175 °C for 1 h. Then, 3 mL polyvinyl pyrrolidone (PVP) (0.375 mol/L) and AgNO_3_ (0.25 mol/L) was added at 175 °C for another 45 min [27]. After cooling, the precipitates were collected and dissolved in ethanol for use.

### 2.2. Preparation of the Sample

The glass cover slides and silicon wafer were first cleaned in a 1 mol/L NaOH solution for 20 min. Subsequently, ITO, FTO, glass, and silicon slides were cleaned using deionized water, acetone, and ethanol), each for 20 min, and blow-dried by clean air. The conductive polymer P3HT was dissolved in chlorobenzene at a concentration of 5 mg/mL with continuously stirring in a water bath at 80 °C for an hour. The PCPDTBT was dissolved in chloroform at a concentration of 5 mg/mL with stirring at 70 °C for an hour. Ag NPs were first sonicated for 10 min to disperse them uniformly without aggregations, and then spin coated onto the glass, silicon, FTO, and ITO glass substrate at 4000 rpm for 30 s (in air). Samples of layers of the conductive polymers P3HT/PCPDTBT on ITO glasses substrates were prepared by spin coating at 3000 rpm for 60 s. Detailed AFM test conditions and principles are presented in the supporting information.

## 3. Results

**Figure 1:**
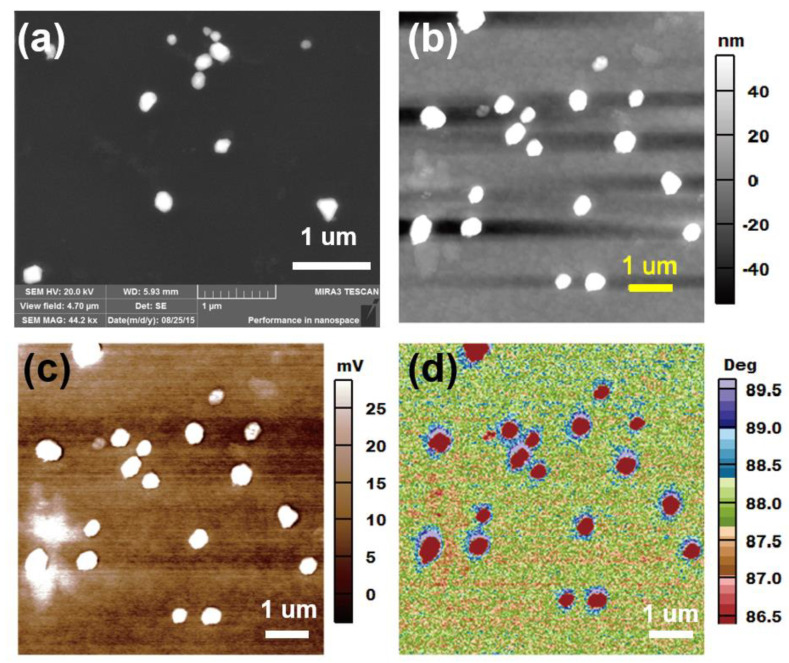
(**a**) A scanning electron microscope (SEM) image of Ag nanoparticles (NPs); (**b**) Atomic force microscopy (AFM) topographic; (**c**) Surface potential; (**d**) Electrostatic force microscopy (EFM) image of the Ag NPs/poly[2,6-(4,4-bis-(2-ethylhexyl)-4*H*-cyclopenta[2,1-b;3,4-bʹ]dithiophene)-alt-4,7-(2,1,3-benzothiadiazole)] (PCPDTBT) sample at a wavelength of 488 nm. V_EFM_ = 6 V. Scale bar: 1 µm.

Both scanning electron microscope (SEM) (Figure 1a) and AFM (Figure 1b) topography were used to characterize the size of these particles. In SEM images, most of the Ag NPs appear spherical. Energy-dispersive X-ray spectroscopy (EDS) was simultaneously conducted; the results indicate that the Ag NPs have been successfully synthesized (Figure S2a). The morphology of Ag NPs covered with conductive PCPDTBT were tested via AFM. The SKPM and EFM images of the sample were simultaneously obtained while the AFM topography of the Ag NPs/PCPDTBT samples are being imaged, as shown in Figure 1c,d, respectively. In both SKPM and EFM images, the Ag NPs are observed to have similar sizes, which indicates that the SKPM (Figure 1c) and EFM (Figure 1d) resolution matches that of the AFM topography (Figure 1b). This reflects the high resolution and accuracy of the data.

**Figure 2:**
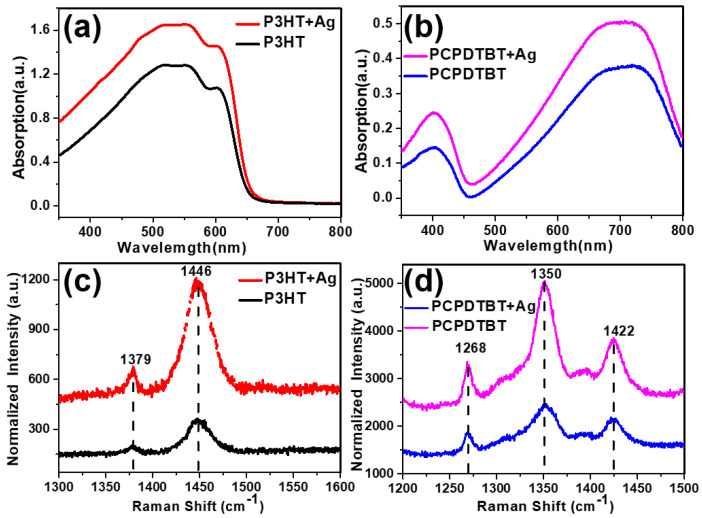
(**a**) The absorbance spectra of films of poly(3-hexylthiophene-2,5-diyl) (P3HT) and P3HT/Ag composite films; (**b**) Raman spectra of films of P3HT and P3HT/Ag composite films; (**c**) The absorbance spectra of films of PCPDTBT, and PCPDTBT/Ag composite films; (**d**) Raman spectra of films of PCPDTBT, and PCPDTBT/Ag composite films.

The UV tests are conducted in order to better illustrate the optical properties of the film formed by the polymer (P3HT/PCPDTBT) and Ag NPs. The absorption spectra of all the samples in the solution are measured before spin-coating them onto the substrates. The absorption of Ag NPs in ethyl alcohol shows a broad band peak at 450 nm (Figure S2b), corresponding to the absorption of the surface plasmon of Ag NPs. The peak frequency is the surface plasmon resonance frequency (*ω*_p_) [28,29]. The absorption spectrum shown in Figure 2a is first used to identify the order of the P3HT molecule. The ordered P3HT showed a significant shoulder at ~600 nm, i.e., P3HT exhibits a broad absorption band, which indicates a wide range of π electron delocalization [20,30]. This structure can be attributed to interchain absorption from highly ordered domains. Therefore, the strength of this signal depends on the regularity of the polymer domain and the accumulation of P3HT chains. Figure 2b shows the absorption spectrum of the PCPDTBT film at a wavelength of 350–800 nm. The absorption spectra show two major broad peaks, at ~410 nm and ~715 nm; this is consistent with previous studies [31]. The PCPDTBT polymer chain includes the donor unit cyclopentadithiophene (CPDT) and acceptor unit benzothiadiazole (BT), which are responsible for absorbing shorter and longer wavelengths, respectively [32]. This results in different optical responses to the different penetration, morphology, and distribution of nanostructures. It is observed that the composite conductive polymer and Ag NP film exhibited the same absorption position as the original P3HT/PCPDTBT; however, the absorption intensity of the composite film has increased significantly. This indicates that Ag NPs have not changed the molecular structure of the polymer. On the contrary, the composite film has a better light absorption due to the unique plasmon effect of the Ag NPs.

We measured the vibration mode peak of the Raman displacement to better explain whether the addition of Ag particles changes the structure of the polymer. Raman spectroscopy is excited at a wavelength of 532 nm. Figure 2c depicts the Raman results for P3HT and the composite film of P3HT and Ag particles. The π-π stacking of the P3HT chain can change the vibration in the conjugate system due to coupling. Therefore, the Raman spectrum of the P3HT film has two typical peaks, at approximately 1379 cm^−1^ and 1446 cm^−1^. This is in accordance with the results of the study reported in [33]. The two main Raman peaks are usually attributed to the stretching of the C-C and C=C rings of the thiophene unit. The C-C and C=C intensity ratios are sensitive to the chain flatness and conjugation length of the P3HT crystal [22]. When mixed with conductive polymers, the surface plasmon of Ag NPs continues to exist can enhance the vibration absorption of the polymer. Compared with the only P3HT film, the Raman spectrum of the P3HT film near Ag NPs shows a high degree of data similarity, as shown in Figure 2c (red line).

Figure 2c shows that the Ag NPs did not change the chemical structure of the P3HT polymer. The Raman intensity became stronger in the presence of Ag NPs owing to the enhancement caused by their surface plasmon. Because the laser spot used in Raman spectroscopy is approximately a few microns, which is much larger than the size of Ag NPs, the increase can only be caused by Ag NPs. The corresponding Raman spectra are also obtained for PCPDTBT and its composite film with Ag NPs in order to verify the accuracy of this conclusion. The enhancements of Raman intensity are observed in the composite conductive polymer PCPDTBT and Ag NPs films (Figure 2d). The results show the Raman spectrum of the PCPDTBT and Ag composite film: the three main Raman peaks are at 1268 cm^−1^, 1350 cm^−1^, and 1422 cm^−1^, collected at wavelengths of 1200–1500 nm [34]. The PCPDTBT conjugated polymer containing two units, PDT (electron-donating unit) and BT (electron-accepting unit), shows a strong polymer/fullerene interaction, which indicates an effective light-induced charge transfer [35]. It can be seen from Figure 2c,d that the Raman strength of the composite film continues to be still stronger than that of the polymer-only film. This is caused by the surface enhanced Raman scattering effect (SERS).

The absorption and resonance Raman spectroscopy result of the conductive polymer P3HT/PCPDTBT and Ag NP composite film show no significant changes, except for an intensity enhancement, thus demonstrating that Ag NPs and conductive polymer composite films significantly increase plasmon and that the surface plasmon of Ag NPs is a near-field effect. The presence of Ag NPs enhances the local electromagnetic field, without significantly affecting the structure.

**Figure 3:**
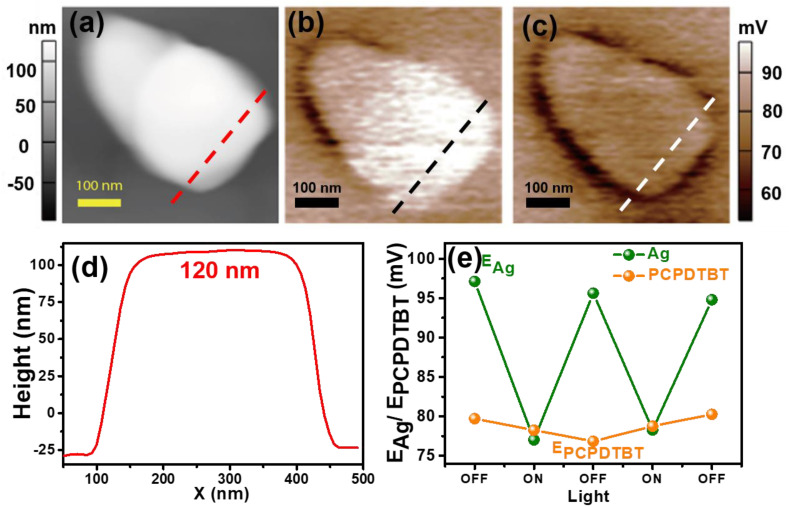
(**a**) AFM topographic of Ag NPs on PCPDTBT films; (**b**) Surface potential AFM images of an Ag nanoparticle on a PCPDTBT film without light excitation and (**c**) with light excitation at a wavelength of 488 nm; (**d**) Table 120 nm; (**e**) The light-modulated surface potential of the Ag nanoparticle on PCPDTBT films.

**Figure 4:**
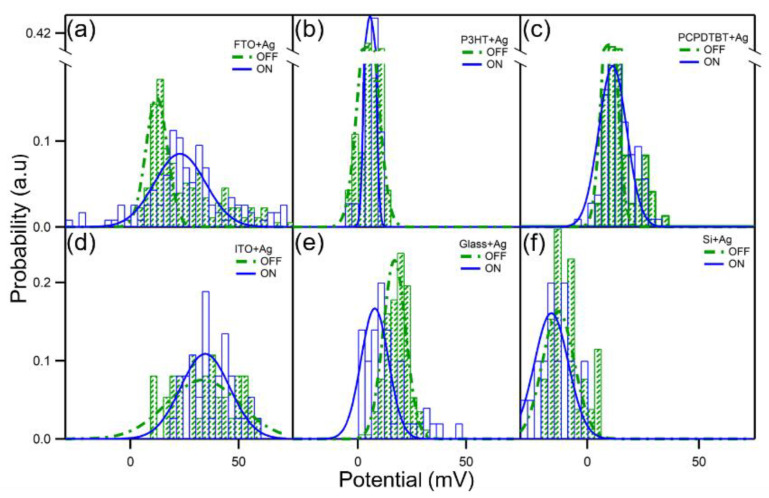
The light-modulated surface potential of Ag NPs on various substrates. The histograms of the surface potential measured in Scanning Kelvin probe microscopy (SKPM) on (**a**) fluorine-doped tin oxide (FTO); (**b**) P3HT; (**c**) PCPDTBT; (**d**) indium tin oxide (ITO); (**e**) glass; (**f**) silicon. On FTO, ITO, P3HT, or glass, a 488 nm laser light is used to illuminate the Ag NPs; on PCPDTBT, both the laser light and light from a white light lamp (solid-state intensity control at 80%) are used; on silicon, only the white light is used.

The AFM morphology of Ag Ag NPs on PCPDTBT films is shown in Figure 3a. The Ag NPs size is close to 120 nm, as shown in Figure 3d. Figure 4b shows he surface potential of the Ag-PCPDTBT composite without light excitation. Figure 3c displays the surface potential when irradiated at a wavelength of 488 nm at the same location. The conductive polymer is completely covered with Ag NPs, i.e., the increased potential of the film is not sufficient to block the charge separation that occurs at the interface of the Ag-PCPDTBT composite material. In other words, the measured surface potential directly reflects the separation and recombination of light-induced charges at the interface. It can be seen from the SKPM images without light (Figure 3b) that the potential of the Ag NPs is larger than the surrounding potential. The Ag NPs show a more positive surface potential, as indicated by a brighter area. The average surface potentials of Ag NPs and polymer PCPDTBT are 96 ± 3 mV and 79 ± 2 mV, respectively. In addition, the surface potential profiles extracted from the location of dotted lines in Figure 3b,c (Figure S3a,b) largely reflect the changes in surface potential of the Ag NPs and polymer PCPDTBTB after illumination, along with some small discrepancies (Figure S3a,b). The difference in the surface potential between the Ag NPs and PCPDTBT films without light is ~26 mV. When light is irradiated, the surface potential of the Ag NPs changes significantly. Under light, the brighter areas become darker (negative surface potential), without any change in appearance. The average surface potential of Ag NPs and polymer PCPDTBT at this time are 77 ± 2 mV and 78 ± 3 mV, respectively (Figure 3c). The surface potential of Ag NPs will be significantly reduced, while the surface potential of PCPDTBT has no obvious change. The change in the surface potential difference is only a decrease of a few millivolts in most cases and it can be attributed to the separation of charges occurring at the Ag/PCPDTBT interface. The negative change in potential is explained in terms of the transfer of excited electrons in the PCPDTBT to Ag.

The separation and transfer of charges at the interface of composite materials are most important in the study of composite photovoltaic materials. After illumination, charge separation of the composite film (Ag-PCPDTBT) is clearly observed and, therefore, further proof of charge recombination at the interface is needed. In the SKPM test, the separation of charge transfer and recombination at the interface is demonstrated by the light alternately turning on/off. The condition of light modulation is to change state every 16 min; this is because the scan time is 16 min. Under light modulation, the bright-dark alternating phenomenon of Ag NPs can be clearly seen via SKPM, i.e., Ag NPs under modulating light showed significant changes. The data are presented in Figure 3e, demonstrating that the electron-hole pair separates and recombines at the interface and the process is reversible.

From the above results, the SKPM mode can directly measure the charge separation of Ag NPs and conduct polymers at the interface. Notably, this process is not observed for all Ag and polymer interfaces. At the interface between a large number of Ag particles and polymer P3HT, this phenomenon is not directly observed via SKPM. Similarly, whether the substrate will have an impact also requires verification and exclusion. The surface potentials of a large number of Ag NPs at different interfaces affected by light are shown in Figure 4 in order to better illustrate the experimental results. The mean and variance of the Gaussian distribution are summarized in Table S1. In the experiment, not only the surface potentials of Ag NPs affected by light at the interface with the polymer but also the surface potentials of Ag NPs on the ITO, FTO, Glass, and Si substrate are considered. The composite film in the experiment is spin-coated onto the ITO substrate, and the test of different substrates are mainly conducted to explain whether the substrate played a role or it has the potential to play a guiding role in future experiments.

According to the results of the histograms presented in Figure 4 and Table S1, it is clear that the relative surface potential of Ag NPs is different for different substrates. It can be seen from the data that the change in the Ag NPs with respect to the surface potential, with the light OFF-ON, is within the error range, except for glass and FTO substrates. On glass substrates, a possible cause of the change in the relative surface potential of the Ag NPs under light is the non-conductivity of glass. In the SKPM test, all of the samples are grounded. Glass is not a good conductor and, thus, more accurate data cannot be obtained during the SKPM test. A more conductive sample results in more authentic and accurate SKPM test results. FTO has better conductivity than glass but it is thicker. This relative thickness is a possible reason why the relative surface potential of Ag particles causes changes after the irradiation with light. Compared to ITO, it is not easy to ground FTO, and the circuit has poor conductivity; this will also have a certain impact on the test results. The silicon substrate is opaque and the sample is irradiated with a 488 nm laser through the bottom; thus, only white light is used in the test. The results show that the potential has not significantly changed. When in the dark state, the average surface potentials of Ag NPs on ITO, and conductive polymer PCPDTBT, and P3HT are 34 ± 5 mV, 10 ± 1 mV, and 5 ± 1 mV, respectively. The average surface potentials of Ag NPs on ITO, conductive polymer PCPDTBT, and P3HT under light are 35 ± 3 mV, 11 ± 1 mV, and 6 ± 1 mV, respectively. Therefore, it is fully dismissed that the conductive substrate may play a role in the charge separation and transfer of the polymer and Ag NPs.

The charge separation at different composite film interfaces cannot be fully observed via SKPM. Therefore, a more intuitive and quantitative method, EFM, is used in order to dynamically study the photoelectric conversion process in these nanocomposite films from the micro/nanoscale, i.e., EFM is used to extract quantitative charge information on the samples [21,26,36,37]. By changing the amplitude and phase of the cantilever of the conductive probe, which is not grounded during the EFM test, an image of the corresponding long-range force can be obtained [22].

EFM images of charged Ag NPs on the conductive polymer PCPDTBT for different biases (6 V, 3 V, 0 V, −3 V, and −6 V) applied to the tip are shown in the supporting information, under a reversed bias from 6 V to −6 V (see Figure S4). Here, the EFM phase images are acquired at a scan rate of 0.5–1 Hz. The phase shift due to the electrostatic force at different voltages is measured [36,38,39]. It is noted that, for Asylum Research MFP-3D, the phase-force response is in contrast to that commonly reported in the literature [26,38]. Therefore, the phase shift, as a function of applied V_EFM_, can be fitted by the following equation: $∆\emptyset =A\left(V_{\mathrm{EFM}}-V_{\mathrm{CPD}}\right)+B\left(V_{\mathrm{EFM}}-V_{\mathrm{CPD}}\right)$ ^2^. Here, ∆∅ is the phase shift of the resonant peak [36]. $V_{\mathrm{EFM}}$ and $V_{\mathrm{CPD}}$ are the applied DC voltage and surface potential of the sample, respectively. At $V_{\mathrm{CPD}}=V_{\mathrm{EFM}}$, the phase shift is equal to zero. A and B are the fitting parameters that are defined as [25]: $A=\left[Q/\left(kd^{2}\right)\right]q$; $B=-\left(Q/k\right)\left[\left(3\alpha \right)/z^{4}\right]$. Here, Q is the quality factor, k is the spring constant, d is the lift height, and α is the electric polarizability. In the testing process, only the same type of conductive probe is used and not the same conductive probe. Therefore, the d value is constant, the values of the k and d will be different. In this work, *k* = 1–3 N/m (nominally 2.8 N/m), Q = 180–200, and d = 100 nm. The charges are computed using coefficients A and B, obtained via the above equations. Formula A is used to calculate the actual charge, according to the Q value measured each time.

**Figure 5:**
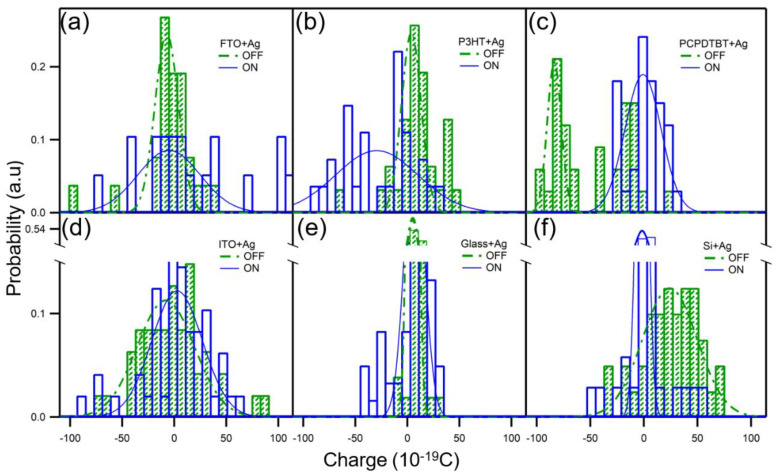
The light-modulated surface charge of Ag NPs on various substrates. The histograms of the surface charge measured in electrostatic force microscopy (EFM) on (**a**) fluorine-doped tin oxide (FTO); (**b**) P3HT; (**c**) PCPDTBT; (**d**) indium tin oxide (ITO); (**e**) glass; (**f**) silicon. On FTO, ITO, P3HT, or glass, a 488 nm laser light is used to illuminate the Ag NPs; on PCPDTBT, both the laser light and light from a white light lamp (solid-state intensity control at 80%) are used; on silicon, only the white light is used.

The surface charge of Ag NPs on the composite film formed with the polymer is measured and the surface charge of Ag NPs on the other four substrates are obtained via EFM. The number of surface charges of Ag NPs on different substrates is quantified and displayed using a histogram, as shown Figure 5. As the statistical values of SKPM, the surface charge of the Ag NPs and the measured data are listed in Table 1. After the composite Ag and polymer (PCPDTBT/P3HT) film is exposed to UV light, the surface charge of the Ag NPs will change significantly. Although the change in charge cannot be discerned by the surface potential, a difference is observed in the EFM results. The average surface charge of Ag NPs on conductive polymer PCPDTBT and P3HT with the lights-off are −84 ± 3.9 and 3.8 ± 2.4 10^−19^ nC, respectively. The average surface charge of Ag NPs on conductive polymer PCPDTBT and P3HT under light are −0.83 ± 4.9 and −29 ± 2.1 10^−19^ nC, respectively. Because coefficient A has both positive and negative sign in the fitting result, the calculated charge also includes positive and negative signs. There is still uncertainty regarding the physical meaning of the sign. Therefore, the calculated charge cannot be accurately expressed as an electron or a hole.

Although the changes in the surface potential cannot be distinguished from the results, the quantitative-charge results of Ag NPs in composite Ag-PCPDTBT and Ag-P3HT films are in contrast to each other. The amount of change in the Ag charge is significantly higher in the Ag-PCPDTBT film than in the Ag-P3HT film. The change in quantitative charge of the Ag NPs on other substrates (Table 1) after irradiation with light is far from being comparable to that on Ag-polymer films. This proves that charge transfer predominantly occurs at the interface between Ag and the polymer. From the SKPM results, it can be seen that the charge is transferred from the PCPDTBT to the Ag NPs in the Ag-PCPDTBT film; however, it is not known how the charge is transferred. From the analysis of the EFM results, it is found that a contrasting change in charge occurs in the Ag-P3HT and Ag-PCPDTBT films. Therefore, it can be speculated that electrons are injected from the resonant Ag NPs to the polymer P3HT through Au excitation by light.

**Figure 6:**
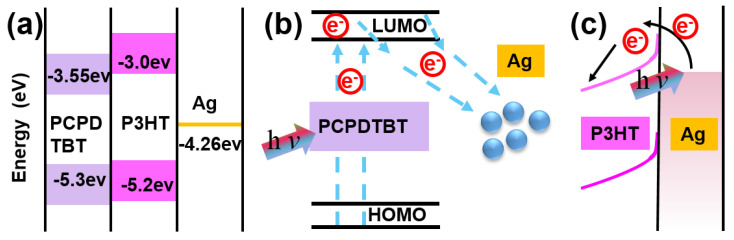
(**a**) Band gap diagram of PCPDTBT and P3HT and the Femi levels of Ag; (**b**) illustration of charge separation on the Ag-PCPDTBT film under light; (**c**) schematic diagram for describing electron transfer for the Ag-P3HT system.

After the SKPM and EFM results are obtained, photo-induced charge transfer is demonstrated in Ag-P3HT and Ag-PCPDTBT films. Figure 6a–c provide a better understanding of the microscopic charge transfer process at the interface. Figure 6a shows a detailed materials characterization concerning the energy gap [40,41,42]. In this graph, The HOMO (highest occupied molecular orbital) and LUMO (lowest unoccupied molecular orbital) energies of the conducting polymer (P3HT/PCPDTBT) and the Femi levels of Ag are clearly displayed. It can be seen from Figure 6 that the LUMO value of PCPDTBT is lower than that of P3HT and the band gap of the former is also smaller than that of the latter. When the conductive polymer is in contact with Ag, the band that formed at the interface between PCPDTBT and Ag is less curved than that between P3HT and Ag. Therefore, PCPDTBT with Ag better facilitates charge transfer than P3HT with Ag.

With the help of Figure 6b, the charge transfer between Ag NPs and PCPDTBT can be more clearly understood. The conductive polymer PCPDTBT is excited by light, forming excitons and generating an electron-hole bound charge pairs after dissociation. When the charge pair migrates to the PCPDTBT and Ag heterojunction, charge transfer occurs and electrons are injected into Ag, whilst holes are retained in PCPDTBT [23], i.e., electrons of the HOMO are excited to the LUMO by the light and are subsequently transferred to the Ag NPs. In the P3HT-Ag (Figure 6c), Ag NPs will cause local surface plasmon resonance under the influence of light. After the conducting electrons in the Ag NPs obtain the energy of the incident light, the energetic electrons will jump to the Fermi level of Ag and be on the surface of the Ag NPs. The charge is then quickly injected into the LUMO of the polymer P3HT; i.e., electrons in plasmon resonance Ag are injected into P3HT LUMO by light and migrate into P3HT. Under the 488 nm laser, PCPDTBT gets excited more easily than P3HT, according to the absorption spectrum. This shows that there is also plasma-induced resonance energy transfer in the Ag-PCPDTBT system, but it is not the predominant factor. The change in surface charge in the polymer PCPDTBT is larger than that in P3HT, which facilitates better charge separation at the interface. These results are also consistent with the charge transfer observed and quantified via SKPM and EFM, respectively.

## 4. Conclusions

In summary, this study used a combination of SKPM and laser irradiation to monitor the photo-induced charge transfer between Ag and a polymer. In the experiment, the transfer of charge from the polymer PCPDTBT to the Ag NPs was observed on a nanoscale via SKPM. The combination of EFM and laser irradiation was used to calculate the photo-induced surface charge on the surface of Ag NPs in Ag-PCPDTBT and Ag-P3HT systems. When the plasmonic Ag NPs were selectively excited with light, electron injection from the resonant Ag NPs to P3HT was evident, as opposed to the case of the PCPDTBT excitation by light. Similarly, from the surface potentials of Ag NPs on different substrates and the change in charge upon illumination, it was determined that charge transfer occurs at the interface of the Ag-PCPDTBT/P3HT system. SKPM and EFM will provide good guidance for optimizing participation in precious-metal photovoltaic solar systems at the nanoscale.

## Figures and Tables

**Table 1:** The average charge number of a series of Ag NPs on different substrates.

Samples	Charge with Light-Off (10^−19^ nC)	Charge with Light-On (10^−19^ nC)
FTO	−7.2 ± 2.1	−5.1 ± 1.7
P3HT	2.8 ± 2.4	−32 ± 2.1
PCPDTBT	−84 ± 3.9	−0.00 ± 4.9
ITO	−8.1 ± 6.1	2.7 ± 7.1
Glass	5.2 ± 0.2	6.8 ± 1.5
Si	21 ± 3.9	−3.7 ± 0.54

## Supplementary Materials

The following are available online at https://www.mdpi.com/2079-4991/10/9/1819/s1, Figure S1: A scheme of light-modulated EFM and SKPM, Figure S2: (a) EDS results of Ag NPs; (b) Absorbance spectra of films of pure Ag NPs, Figure S3: Surface potential of Ag NPs extracted from Figure 4b,c, Figure S4: (a) AFM image of Ag-PCPDTBT film; (b–f) EFM images Ag-PCPDTBT film under a bias voltage of (b) 6 V; (c) 3 V; (d) 0 V; (e) −3 V; (f) −6 V, Table S1: The average surface potential of Ag nanoparticles on different substrate at different light conditions as listed in Figure 4.
