# Supplementary material for: Atomic Force Microscope Study of Ag-Conduct Polymer Hybrid Films: Evidence for Light-Induced Charge Separation
Source: Nanomaterials (Basel). 2020 Sep 12;10(9):1819. doi: 10.3390/nano10091819 (PMC7558113; doi:10.3390/nano10091819)
Supplement: Supplementary file 1 [file nanomaterials-10-01819-s001.pdf]

nanomaterials

Supplementary Materials

Atomic Force Microscope Study of Ag-Conduct Polymer Hybrid Films: Evidence for Light-Induced Charge Separation

Yinghui Wu, Dong Wang, Jinyuan Liu, Houzhi Cai and Yueqiang Zhang *

Key Laboratory of Optoelectronic Devices and Systems of Ministry of Education and Guangdong Province, College of Physics and Optoelectronic Engineering, Shenzhen University, Shenzhen 518060, China; yinghui@szu.edu.cn (Y.W.); wangdong20@szu.edu.cn (D.W.); ljy@szu.edu.cn (J.L.); hzcai@szu.edu.cn (H.C.)

* Correspondence: yueqiang.zhang@szu.edu.cn; Tel.: +18-(66)-7176566

Experimental Section

Spectroscopic Characterization

The absorption of samples in the wavelength range of 350–750 nm were measured by a UV-visible spectrometer (LAMBDA950, PerkinElmer, Waltham, MA, USA). The Raman scattering spectra measurements were performed by a commercial Micro-Raman spectrometer (DXR2, Thermo, San Francisco, California, USA) with a 532 nm laser, whose polarization was perpendicular to the Ag NPs. In our Raman experiments, the laser power irradiating the sample was measured at 0.1 mW with a 50× objective. The laser light of 488 nm Sapphire LP OEM laser (Sapphire 488-120 FP, Coherent, San Francisco, California, USA), and the sample was illuminated from the bottom through the objective at an intensity of 0.2 W/cm^2. The Fiber-Lite Mi-150 (Dolan Jenner, FIBER-LITE MI-150, Shenzhen, China) is used, and its light intensity is controlled at 80%. The size of Ag NPs is characterized on a silicon wafer by a MERLIN (Carl Zeiss AG, Jena, Germany).

Atomic Force Microscopy (AFM), Scanning Kelvin Probe Microscopy (SKPM), and Electrostatic Force Microscopy (EFM)

All of the AFM-based Experiments were performed in ambient condition using an MFP-3D AFM (MFP-3D-Stand Alone, Asylum Research, Abingdon-on-Thames, UK), and Pt/Cr coated silicon tips (Multi75E-G Budget sensor, Asylum Research, Abingdon-on-Thames , UK) with a spring constant of k ~ 2–5 N/m, a resonance frequency at ~ 60–70 KHz, a tip radius of curvature is 30 nm were used in imaging. The measurement of AFM is carried out at approximately the same temperature (24 °C–28 °C) and humidity (33%–38%). A scan rate of ~0.5–1.0 Hz is used in order to evaluate the light-induced composite film surface potential change of the sample.

SKPM and EFM measurements were both carried out in the two-pass manner. The first pass was used to determine the topography of the surface, and it was done exactly like a standard tapping mode scan line in AFM. The second pass was done by retracing the topography at a constant distance in z direction.

In SKPM and EFM (Figure S1), the tip and the sample interaction could be treated as a parallel plate capacitor, and then the electrostatic force was proportional to the square of the applied voltage. Assuming the voltage between the sample and the probe, and the equivalent capacitance C, the total energy of the system is:

$$U = -\frac{1}{2}C\Delta V^2$$

Taking z as the distance from the sample surface at the normal direction, the electrostatic force on the tip could be expressed as:

$$F = \frac{1}{2}\frac{dC}{dz}V^2$$

Where F is the electrostatic force acting on the tip, V is the potential, C is the capacitance, and z is the distance from the tip to the sample [1,2].

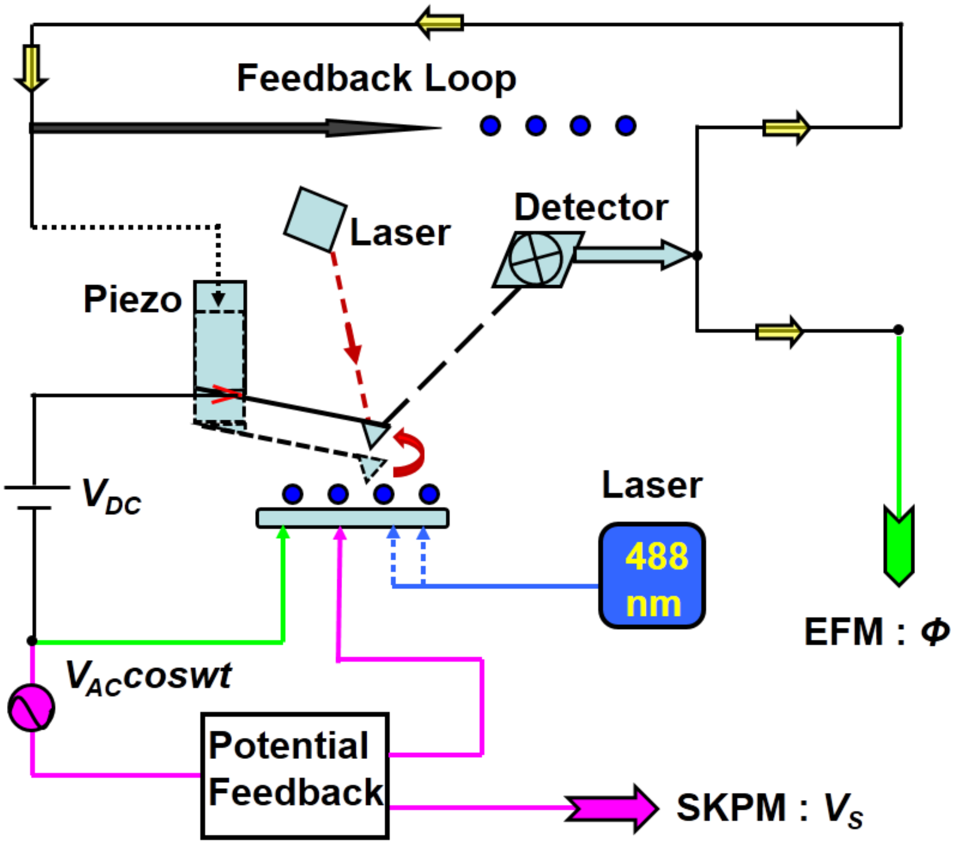

Figure S1. A scheme of light-modulated EFM and SKPM.

In SKPM, an alternating current (AC) voltage and a tunable direct current (DC) voltage were applied between the tip and the substrate during the second pass. The DC voltage was adjusted at each point to cancel the force at the AC frequency and, accordingly, the value was recorded as the potential difference.

In case of SKPM, the voltage between the tip and the sample is:

$$V_{(t)} = V_S - V_{DC} + V_{AC}\sin\omega t \quad (1)$$

$$F_{(t)} = -\frac{1}{2}\frac{dC}{dz} \times V_{(t)}^2$$
$$= \frac{1}{2}\frac{dC}{dz} \times \left[(V_S - V_{DC})^2 - \frac{1}{2}V_{AC}^2\right] + \frac{dC}{dz} \times (V_S - V_{DC}) \times V_{AC}\sin\omega t - \frac{1}{2}\frac{dC}{dz} \times V_{AC}^2\cos 2\omega t \quad (2)$$

Where, $V_{(t)}$ is the tip potential, V_{DC} and V_{AC} are the DC and AC voltage (a frequency of ω) applied between the tip and the substrate, respectively, and V_S is the surface potential of the sample. The vertical electrostatic force is a summary of terms at three frequencies, DC, ω and 2ω, as indicated in Equation (2). According to Equation (2), at the frequency ω, if the V_{DC} was adjusted to zero the force signal, then it should be equal to the surface potential (V_S) of the sample. In SKPM, the V_{DC} is actively adjusted to cancel the signal from the Lock-in amplifier at the frequency ω and recorded as the surface

potential while imaging [3,4]. In SKPM measurement, an offset bias voltage is applied between the sample and needle tip to counteract the electrostatic force between the tip and samples.

In EFM (Figure 1), only a constant DC voltage was applied, and the phase shift of the tip was used to measure the electrostatic force between the tip and the sample. As the tip was raised up a distance from the sample, the major part of force on the cantilever was the long-range tip-sample interactions, e.g., electrostatic force [5,6].

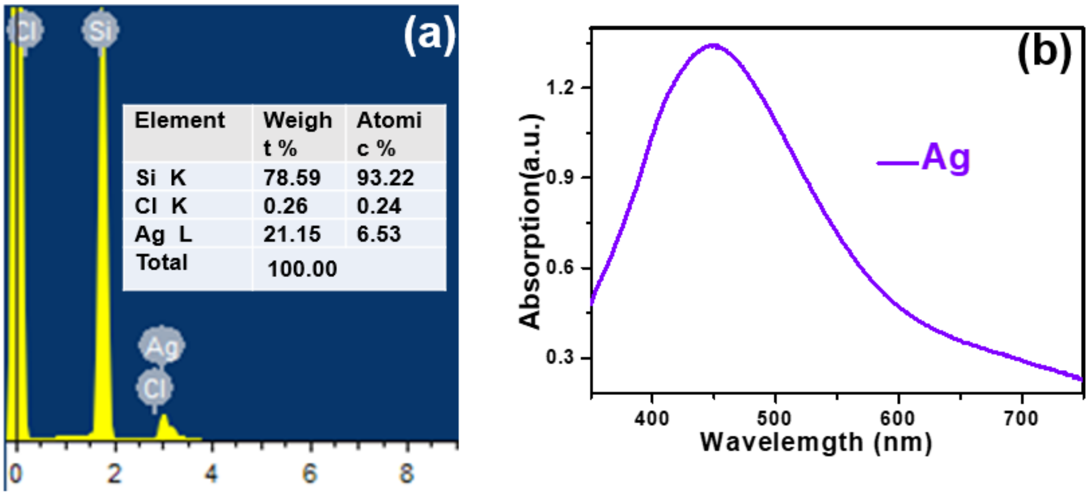

Figure S2. (**a**) EDS results of Ag NPs; (**b**) absorbance spectra of films of pure Ag NPs.

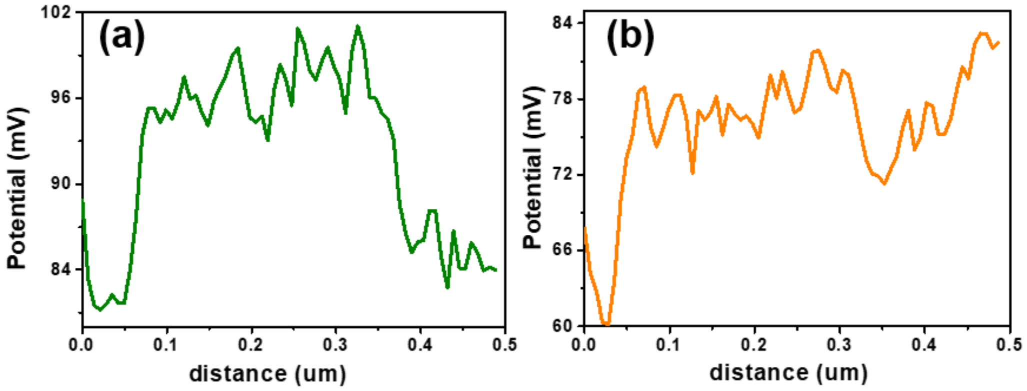

Figure S3. Surface potential of Ag NPs extracted from Figure 4b,c.

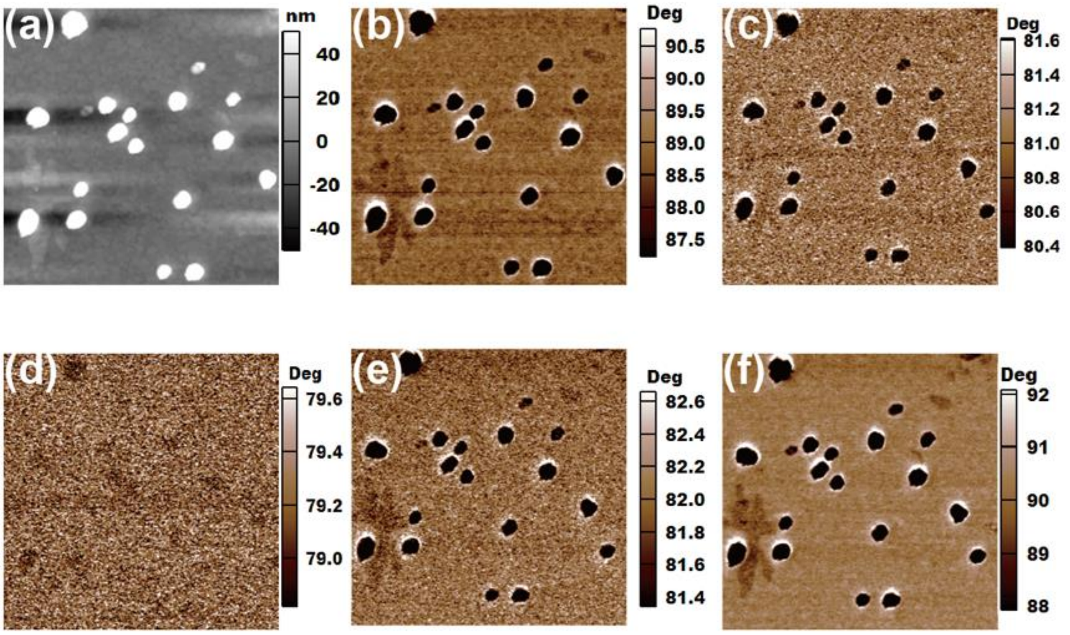

Figure S4. (**a**) AFM image of Ag-PCPDTBT film; (**b**–**f**) EFM images Ag-PCPDTBT film under a bias voltage of (**b**) 6 V; (**c**) 3 V; (**d**) 0 V; (**e**) −3 V; (**f**) −6 V.

Table S1. The average surface potential of a series of Ag NPs on different substrates.

Samples	Potential with Light-Off (mV)	Potential with Light-On (mV)
FTO	12 ± 1	23 ± 3
P3HT	5 ± 1	6 ± 1
PCPDTBT	10 ± 1	11 ± 1
ITO	34 ± 5	35 ± 3
Glass	17 ± 1	8 ± 1
Si	−13 ± 3	−16 ± 2

References

1. Liu, R. Imaging of Photoinduced Interfacial Charge Separation in Conjugated Polymer/Semiconductor Nanocomposites. *J. Phys. Chem. C* **2009**, *113*, 9368–9374.
2. Zhang, L.-P.; Chen, B.; Wu, L.-Z.; Tung, C.-H.; Cao, H; Tanimoto, Y. Remote Activation of the Quadricyclane Group in a Quadricyclane−Steroid−{Dibenzoylmethanatoboron Difluoride} System by Intramolecular Electron Transfer. *J Phys Chem A* **2003**, *107*, 3438–3442.
3. Melitz, W.; Shen, J.; Kummel, A.C.; Lee, S. Kelvin probe force microscopy and its application. *Surf. Sci. Rep.* **2011**, *66*, 1–27.
4. Nonnenmacher, M.; O'Boyle, M.P.; Wickramasinghe, H.K. Kelvin probe force microscopy. *Appl. Phys. Lett.* **1991**, *58*, 2921–2923.
5. Melin, T.; Diesinger, H.; Deresmes, D.; Stiévenard, D. Probing Nanoscale Dipole-Dipole Interactions by Electric Force Microscopy. *Phys. Rev. Lett.* **2004**, *92*, 166101.
6. Mélin, T.; Diesinger, H.; Deresmes, D.; Stiévenard, D. Electric force microscopy of individually charged nanoparticles on conductors: An analytical model for quantitative charge imaging. *Phys. Rev. B* **2004**, *69*, 035321.
